# Establish pre-clinical diagnostic efficacy for parathyroid hormone as a point-of-surgery-testing-device (POST)

**DOI:** 10.1038/s41598-020-75856-2

**Published:** 2020-11-02

**Authors:** Ambalika S. Tanak, Sriram Muthukumar, Ibrahim A. Hashim, Shalini Prasad

**Affiliations:** 1 Department of Bioengineering, The University of Texas at Dallas, 800 W. Campbell Rd. BSB 11, Richardson, TX 75080 USA; 2 EnLiSense LLC, 1813 Audubon Pondway, Allen, TX 75013 USA; 3 Department of Pathology, UT Southwestern Medical Center, Dallas, TX 75390 USA

**Keywords:** Diagnostic markers, Sensors and probes

## Abstract

Measuring the Parathyroid hormone (PTH) levels assists in the investigation and management of patients with parathyroid disorders. Rapid PTH monitoring is a valid tool for accurate assessment intraoperatively. Rapid Electro-Analytical Device (READ) is a point-of-care device that uses impedance change between target and capture probe to assess the PTH concentration in undiluted patient plasma samples. The aim of this work focuses on evaluating the analytical performance of READ platform to Roche analyzer as a prospective clinical validation method. The coefficient of variation (CV) for intra-assay imprecision was < 5% and inter-assay imprecision CV was < 10% for high (942 pg/mL) and low (38.2 pg/mL) PTH concentration. Functional sensitivity defined at 15% CV was 1.9 pg/mL. Results obtained from READ platform correlated well (r = 0.99) with commercially available clinical laboratory method (Roche Diagnostics) to measure PTH concentrations with a turn-around time of less than 15 min. Furthermore, the mean bias of 7.6 pg/mL determined by Bland–Altman analysis, showed good agreement between the two methods. We envision such a sensing system would allow medical practitioners to facilitate targeted interventions, thereby, offering an immediate prognostic approach as the cornerstone to delivering successful treatment for patients suffering from primary hyperparathyroidism.

## Introduction

Parathyroid Hormone (PTH) measurement is essential for the assessment and management of patients with parathyroid gland dysfunction with primary hyperparathyroidism (PHPT), being the third most common endocrine disorder^1^. While most primary hyper parathyroid patients have a single abnormal gland up to 20% of patients will either have multiple adenomas or hyperplasia of all four glands^2^. Surgery is the only curative treatment for PHPT^3^. PHPT surgical procedures can be challenging and carries ambiguity regarding the presence or absence of the disease in a single or multiple hyperplastic glands^4^.

Several imaging techniques provide information about the location of the adenoma as a pre-operative study prior surgery but may not be sensitive to detect multiglandular hyperplasia^5^. Measurement of circulating PTH levels additionally aids in the investigation of calcium disorders^6^. PTH is a single chain 84 amino acids polypeptide produced by the parathyroid gland and in concert with vitamin D and other mediators is responsible for regulating body calcium homeostasis^7,8^. In addition to diagnosis and management, PTH measurement is essential to guide surgical interventions where due to its short half-life (1–3 min) an intraoperative decline in circulating levels indicates successful surgical resection of a hyperfunctioning adenoma^9^. Laboratory-based assays at best take up to 60 min to deliver results from the time sample is received in the central laboratory^10^. During this time, the patient is kept in the operating room while the surgeon waits for results^11^. Additionally, in patients with failed initial surgery, surgical selective thyroid venous catheterization is performed, and resection surgery is repeated several weeks later once laboratory PTH result is available. This highlights the need for a rapid and reliable technique to measure PTH which facilitates point of surgery testing (POST). In addition to the rapid turnaround time, the device must afford specificity for PTH since, the measurement of PTH is complicated by the presence of several molecular forms of the hormone. The intact form being 1–84 amino acids, a mid-molecular form (7–84 amino acid), and a N-truncated form.

Although some assays are termed intact, they also detect the 7–84 fragment, which accumulates in patients with renal dysfunction and thus limits such assays and interpretation. Our previous work established reliable PTH detection in undiluted human serum, plasma and whole blood with a rapid response time^12^. The physicochemical properties of the READ sensor platform captures specific target PTH interaction using electrochemical impedance spectroscopy (EIS) as the detection modality. EIS has been incorporated in many point-of-care sensor applications owing to its label-free approach with the least procedural complexity to acquire results^13–17^. A major advantage of using EIS technique is its ability to capture subtle electrochemical changes by optimizing input parameters. Impedance sensing has already paved its way into clinical entourage such as blood impedance analysis^18^, electrical impedance topography^19^ and electrical impedance myography^20^.

This work focuses on expanding the previously established scope of research for its use as a clinical utility by validating its results with a standard laboratory analyzer (Roche diagnostic) with 40 patient samples^12^. Our aim is to allow surgeons along with their surgical staff to conduct dynamic PTH measurements during resection of the hyperfunctioning gland procedure without the need for a laboratory technician in a convenient and effective manner to facilitate improved patient outcome.

## Results

**Figure 1:**
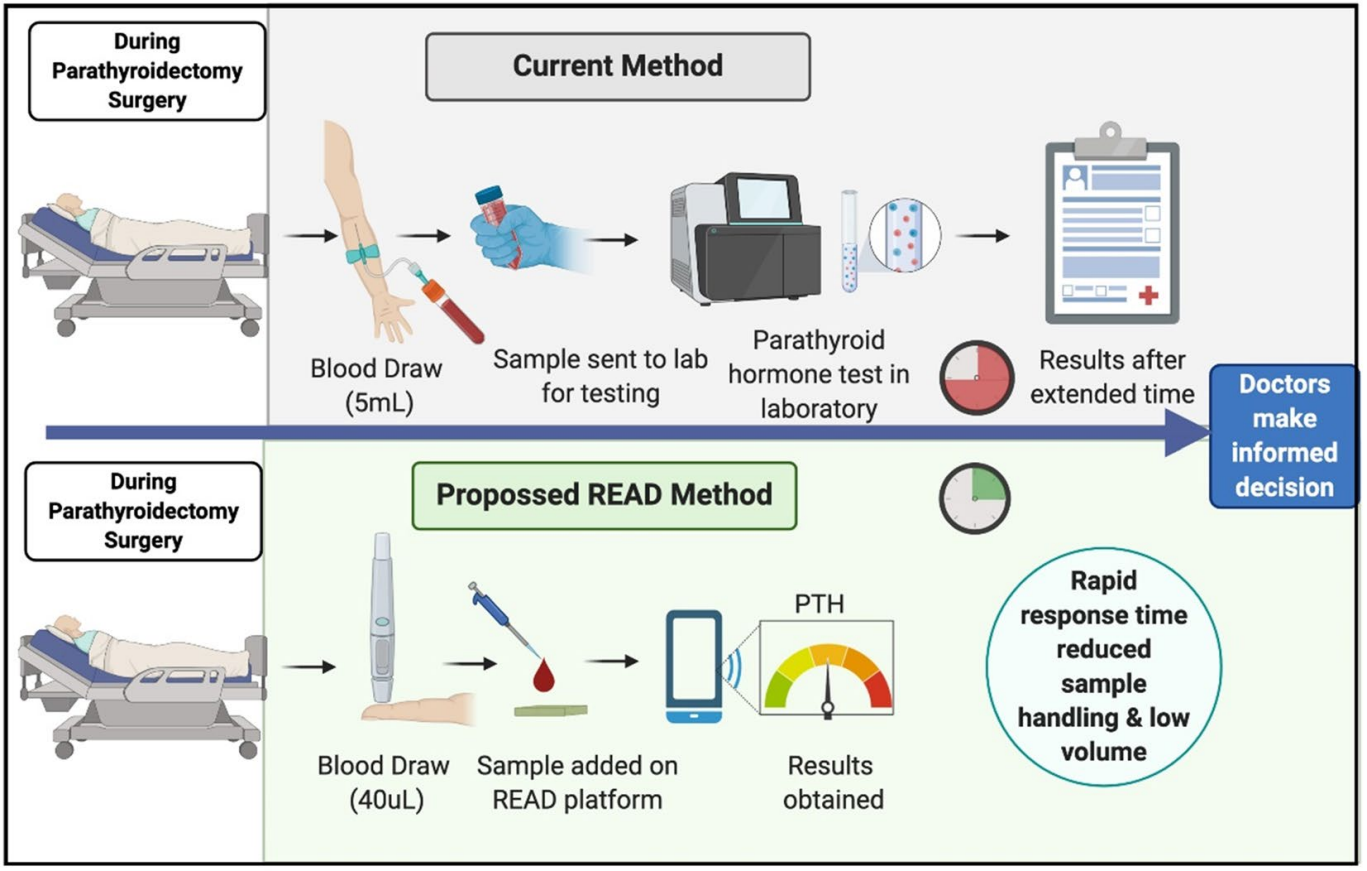
Schematic illustration of use case scenario during parathyroidectomy demonstrating ease of use with READ (represented in lower half) as a point of surgery testing (POST) device. Figure created with BioRender.com.

A use case scenario is illustrated as a schematic diagram in Fig. 1. Top half of the figure represents the current method used during parathyroidectomy surgery where significant time is expended in sample transit and receiving results to confirm PTH levels determining successful operation. The proposed method specified in the lower half of Fig. 1 reveals an enhanced technique that would assist surgeons to determine complete removal of hyperfunctioning parathyroid tissue from the patient during surgery by assessing PTH concentration within 15 min using READ platform.

**Figure 2:**
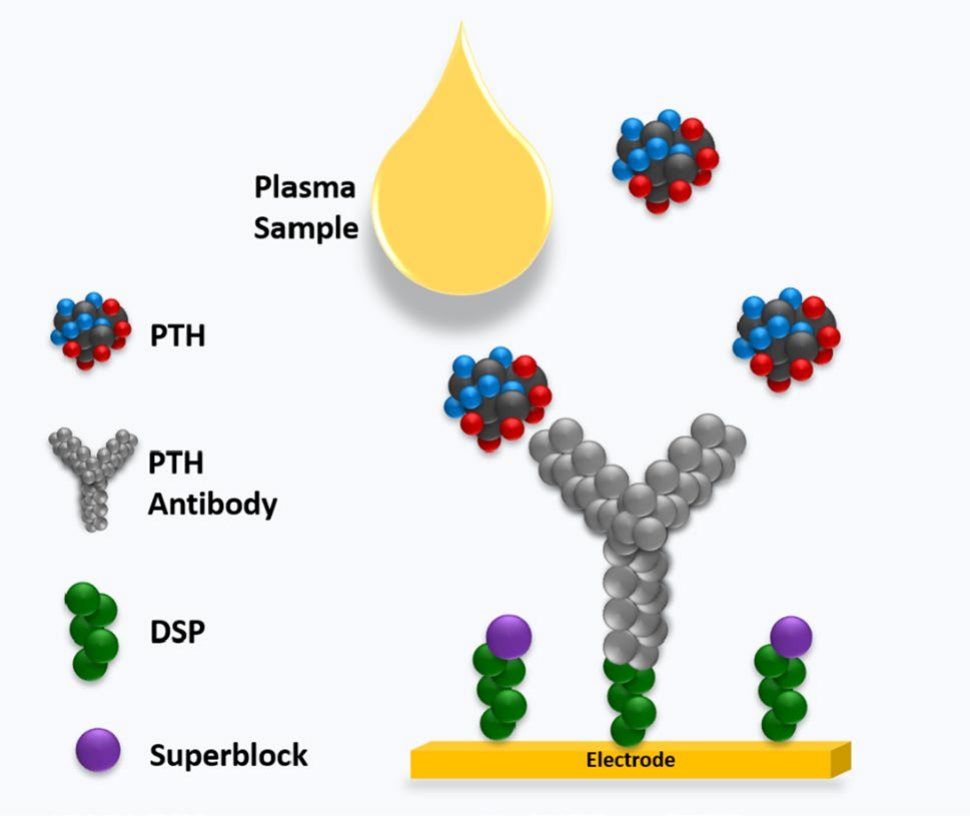
Schematic representation of the Immunoassay built on READ platform.

**Figure 3:**
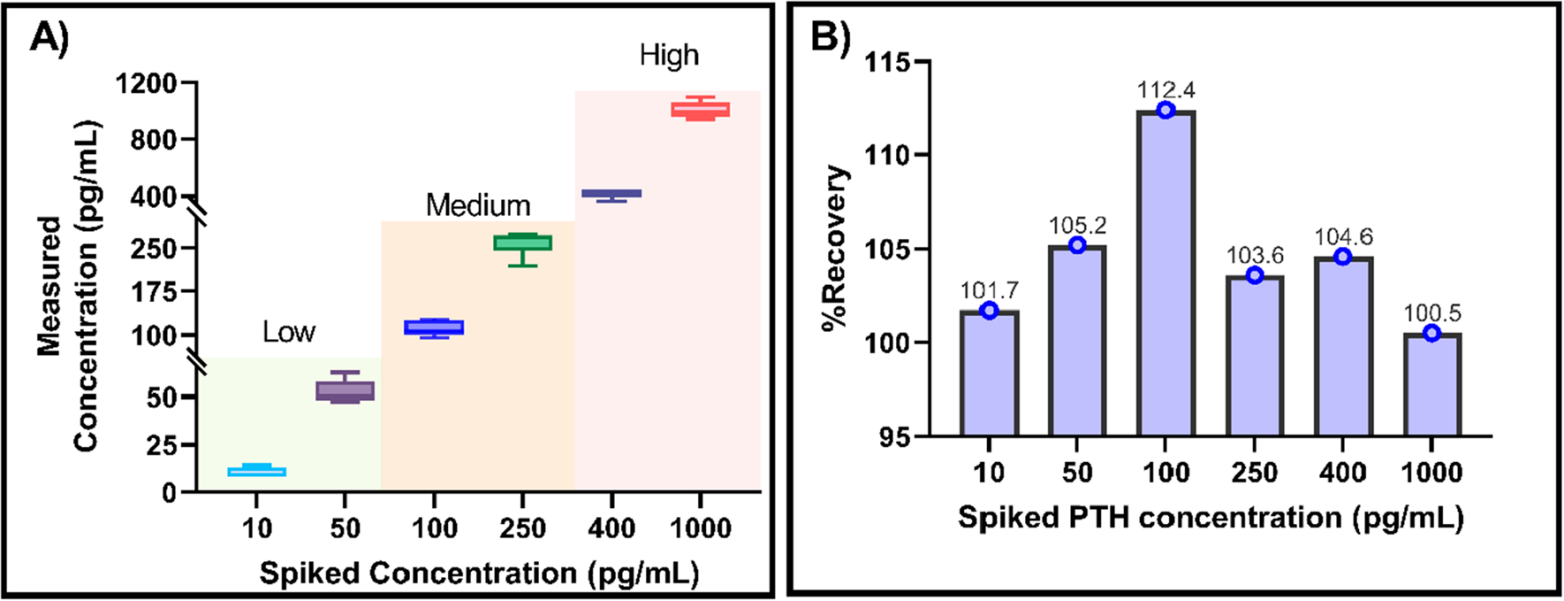
(**A**) Varying PTH concentration (low, medium & high) spiked in plasma samples plotted against measured PTH concentration. (**B**) Recovery analysis for READ platform.

**Figure 4:**
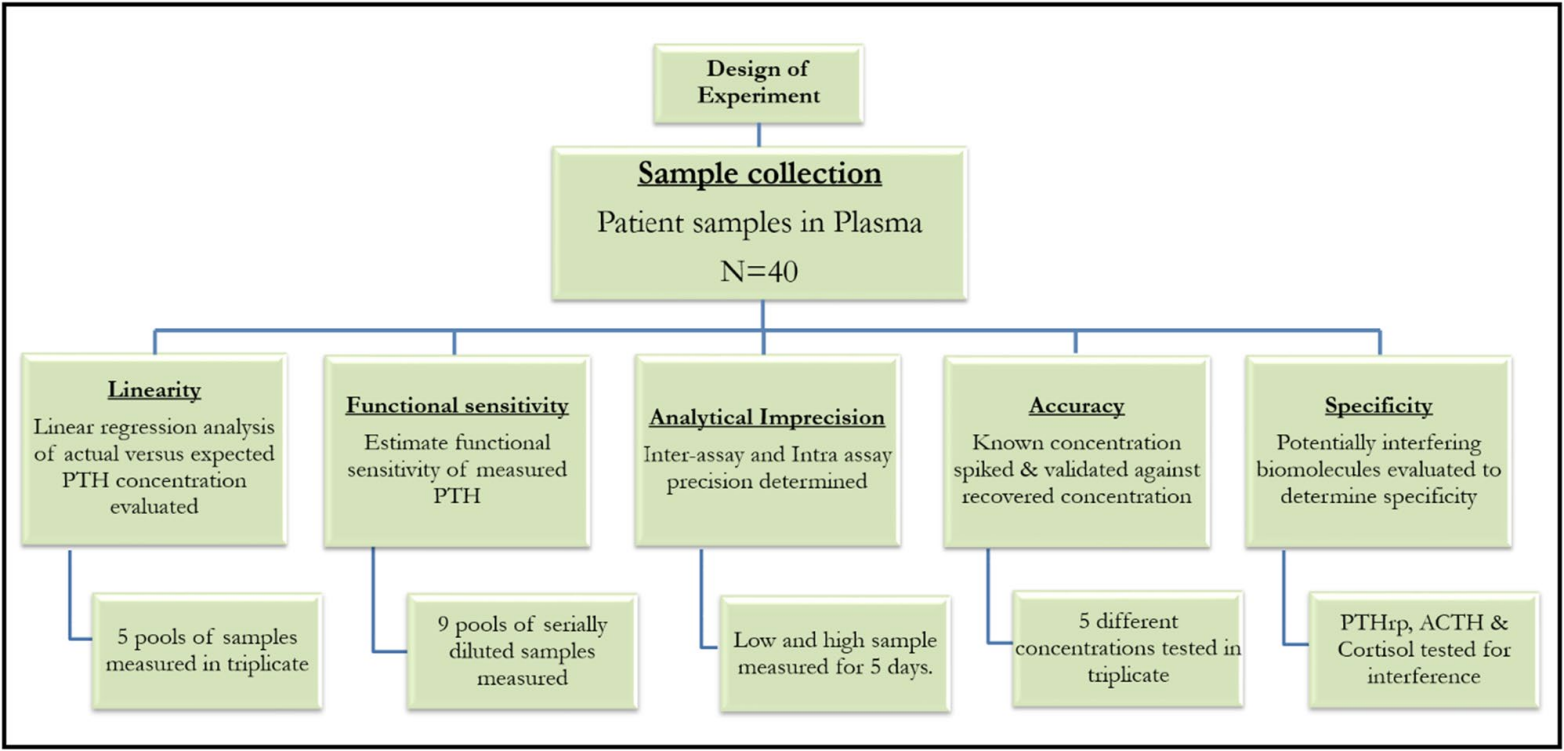
Flow chart describing the design of experiment.

We had previously demonstrated reliable detection of PTH in undiluted human serum, plasma and whole blood as a point of care biosensor using electrochemical impedance spectroscopy. Binding interaction between the target PTH and specific capture probe causes change in impedance occurring at the sensor’s electrode interface. The dose dependent change in impedance quantifies the amount of PTH bound to the specific capture probe. Results demonstrated in the previous work provide foundation for the current work to enable effective validation for clinical implementation. This work extends the scope of READ platform to be established for clinical application. Figure 2 illustrates the schematic representation of the immunoassay developed on READ sensor platform using thiol crosslinker chemistry followed by conjugating specific monoclonal PTH antibody. Calibrated dose response was established for READ platform with varying PTH doses spiked in human plasma. Figure 3A represents the dose response of PTH across a wide dynamic range from 10 to 1000 pg/mL in human plasma samples. Non-overlapping interquartile ranges for each concentration in the box plots portrays the accuracy of the recovered concentrations with respect to its spiked concentrations, indicating minimum variation among replicates. Analysis of variance (ANOVA) showed statistically significant differences between means across low, medium and high concentrations with *p* value < 0.0001 and an R squared of 0.9940. Recovery percentage for each PTH concentration can be seen in Fig. 3B across 6 replicates. PTH concentration recovery ranged from 100 to 112%, with an average recovery of 104.7% ± 4.1, which lies within the acceptable recovery range (80–120% of the expected concentration) as per the Clinical laboratory Standards Institute^21^ (CLSI). Furthermore, highest coefficient of variation (CV) percentage achieved was 11.7%, plotted for each PTH concentration revealed good precision with minimal variation among replicates for READ platform which can be seen in Supplementary Figure 1. Spike and recovered concentrations for PTH have been tabulated in Supplementary Table 1. Recovery analysis confirms the ability of READ platform to reliably measure multiple PTH concentrations in spiked plasma samples. READ platform demonstrated a limit of detection (LoD) of 1 pg/mL with a clinically relevant dynamic range of 1–1000 pg/mL as previous established in our work^12^. The major focus of this study is to analytically validate the performance of READ platform with the Roche lab analyzer. With that reliable READ sensor performance established, analytical validation was evaluated by following the design of experiments illustrated in Fig. 4 for accuracy and reliability to be useful for effective clinical decision making.

### Analytical validation

#### Method comparison

**Figure 5:**
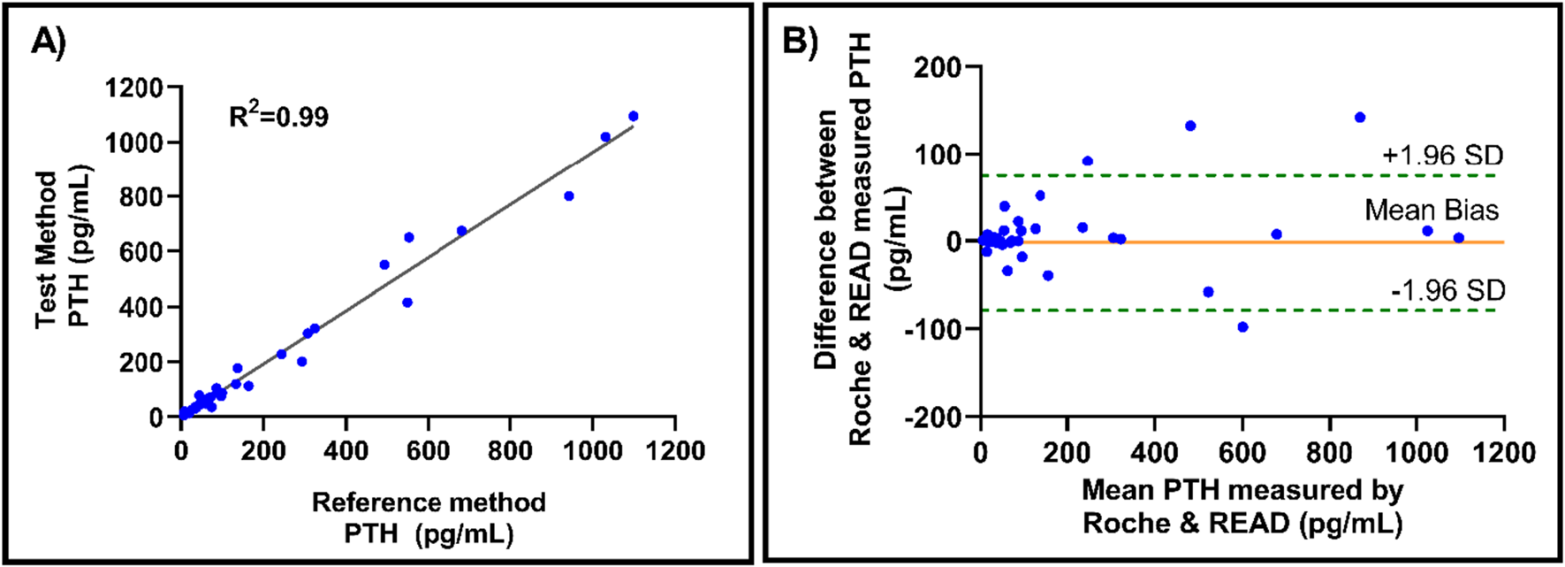
(**A**) Reference method (Roche analyzer) compared with Test method (READ platform) using 40 patient plasma samples with a Pearson’s r = 0.99. (**B**) Bland–Altman plot compares PTH measured by Roche analyzer and READ platform for 40 patient plasma samples. Orange solid line represents mean bias of 7.6 pg/mL. 1.96 SD is represented as green dotted lines computed at 88.07 pg/mL and − 72.77 pg/mL respectively.

The primary goal of method validation is to ensure accuracy of READ platform for the reported results. To clinically validate the performance of READ, PTH concentration was measured in 40 patient samples using Roche analyzer (laboratory standard) and READ platform covering a range from 1 pg/mL to 1050 pg/mL, as represented in Fig. 5A. Correlation along with linear regression analysis for patient plasma samples compared by the two methods had an intercept of 0.0209, 95% confidence interval (CI), 0.91 to 1.007; slope of 0.96, 95% CI, − 15.74 ,15.79 and a Pearson’s r of 0.99. Correlation analysis quantified the degree to which PTH measured by both methods were related with a linear relationship. High correlation does not necessarily imply a good agreement between the two methods, thus, Bland–Altman analysis was performed, as seen in Fig. 5B. In this residual method, difference between two paired measurements was plotted against mean of the two methods. The resulting graph was a scatter plot with x axis represented as the measured mean PTH concentration, while y axis showed difference between the PTH measured using Roche analyzer and READ platform. The bias indicated how series of measurements agreed with the comparative measuring technique. The mean bias between Roche analyzer and READ platform was 7.6 pg/mL. All the sample points were well within the 95% CI (± 1.96 SD) with an exception of 4 points. Bland–Altman analysis confirmed overall agreement between the Roche analyzer and the developed READ platform.

#### Imprecision

Precision was determined by assessing multiple measurements with two patient samples (low and high) within the same day and across 5 days. Precision for READ platform was established in terms of repeatability (intra-assay imprecision) and reproducibility (inter-assay imprecision). The intra-assay and inter-assay imprecision represented as CV for READ was 3% and 8% for low PTH level (38.2 pg/mL) whereas intra-assay & inter-assay imprecision CV for high PTH level (942 pg/mL) was 3% and 10.5% respectively, as seen in Table 1.The CV for both low and high level of PTH samples lie well within the clinical standard practice (< 20%) as per the Clinical and laboratory standards institute guidelines^22,23^.

**Table 1:** Representing the inter-assay and intra-assay variability as measured by READ platform.

Sample	Reported concentration (pg/mL)	Intra-assay CV%	Inter-assay CV%
Low	38.3	3	8
High	942	3	10

#### Functional sensitivity

**Figure 6:**
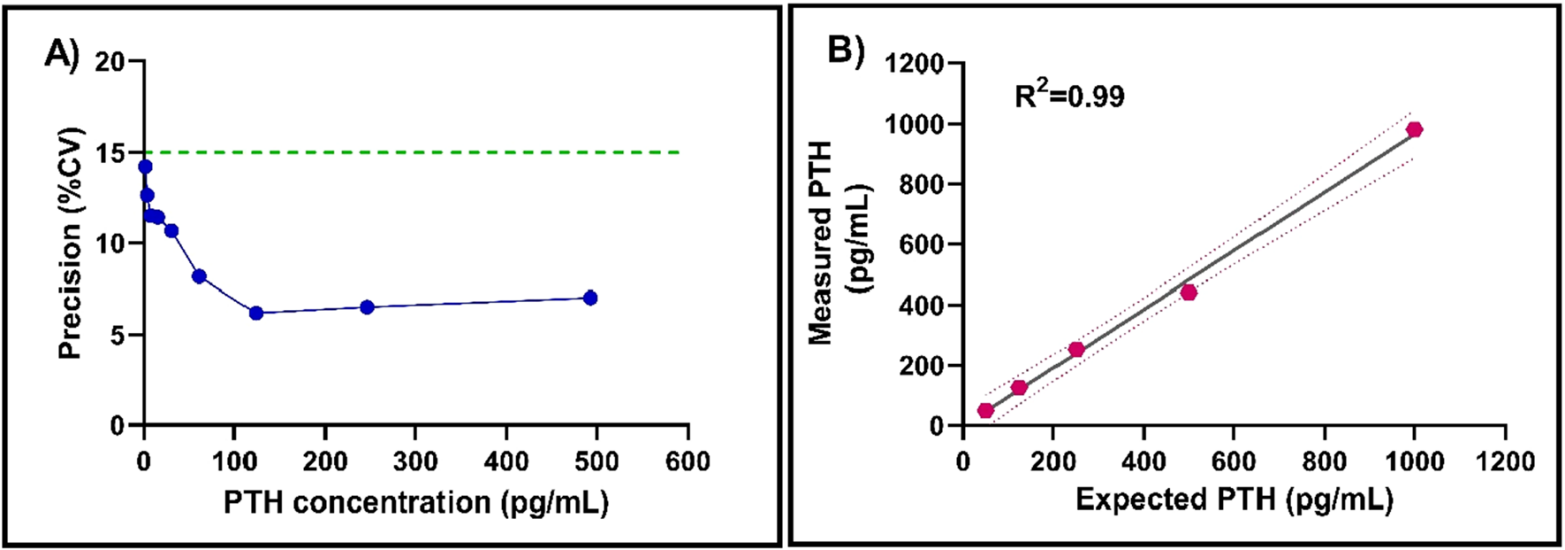
(**A**) Functional sensitivity of READ platform. CV defined at 15% and was 1.96 pg/mL. (**B**) Dilution linearity across 5 PTH concentrations were analyzed with an R^2^ of 0.99 measured with the READ platform.

Functional sensitivity determines the lowest concentration representing clinical usefulness for a given assay. In this work, the functional sensitivity for PTH was defined at 15% CV with a concentration of 1.9 pg/mL as seen in the precision profile represented in Fig. 6A. A CV of 20% is widely accepted for being clinically useful according to CLIA requirements^24^. Consequently, a limit of detection (LoD) of 1.9 pg/mL was achieved for the PTH assay when measured by READ platform. The outcomes of the pre-clinical validations suggest the ability of READ platform to identify low PTH concentrations directly from undiluted patient blood plasma samples, as a POST device with results acquired under 15 min of sampling time.

### Linearity

Linearity of the READ platform was assessed to evaluate the accuracy across PTH working range. Linearity of READ platform was achieved when measured PTH results were directly proportional to the PTH concentration in the test analyte. Furthermore, READ platform demonstrated a linear response for the concentrations tested from 50 to 1000 pg/mL as shown in Fig. 6B with an r^2^ = 0.99.

### Repeatability

**Figure 7:**
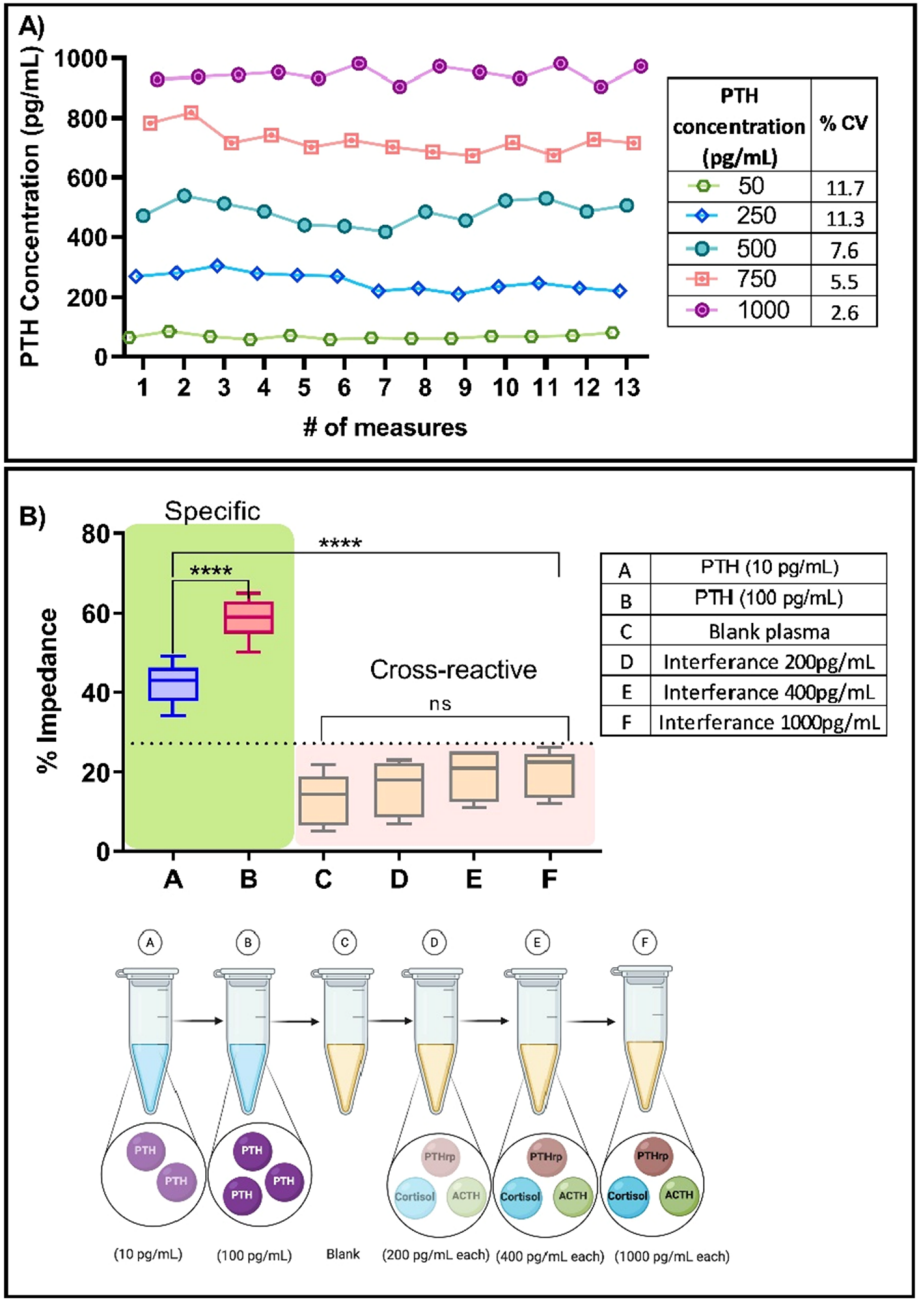
(**A**) Repeatability of READ validated across 13 measurements for 5 patient plasma sample. (**B**) Specificity of READ platform where A represents low (10 pg/mL) concentration of PTH, B represents high (1000 pg/mL) concentration of PTH, C represents blank plasma. D, E and F represents cross-reactive mixture of cortisol, Parathyroid hormone related protein (PTHrp) and Adrenocorticotropic hormone (ACTH) biomolecules in low (200 pg/mL per molecule), medium (400 pg/mL per molecule) and high (1000 pg/mL per molecule) concentrations respectively. Dotted line represents signal threshold. The pictographic representation of cross-reactive mixtures vials is created with BioRender.com.

Repeatability study evaluated the closeness of agreement with multiple measures obtained with READ platform. Figure 7A demonstrates the repeatability performance of READ sensor platform by measuring five patient samples across the quantifiable detection range with 13 measurements performed under similar conditions. The repeatability (%CV) for READ platform with patient samples ranged from 11.7 to 2.6% for 50 pg/mL and 1000 pg/mL of PTH patient concentration in plasma respectively. This showed that results obtained using READ platform were reliable and repeatable.

### Interference

To evaluate selectivity of READ platform towards PTH in the presence of common cross-reacting biomolecules, interreference study was conducted with highest concentration of non-specific molecules like Cortisol, ACTH, and PTHrp. The interference measured from varying non-specific molecules showed no significance when compared with specific PTH dose response. Figure 7B displays specific PTH signal response (A and B) and non-specific response (C, D, E and F) as percentage change in impedance. Specific PTH signal response for the median value as seen in bar A contributes to 43% which is approximately ~ 1.6 times greater than the non-specific response seen in the highest concentration (1000 pg/mL) of the cross-reactive mixture in F that accounts for 26% impedance change. Furthermore, all the remaining cross-reactive mixtures (C, D, E and F) of varying concentrations lie well within the established signal threshold as indicated by the dotted line and hence can be considered as noise. READ platform was able to significantly distinguish specific PTH concentration from cross-reacting molecules of cortisol, PTHrp and ACTH despite being spiked with high concentration of 1000 pg/mL each.

## Discussion

The utility of intraoperative PTH has significantly improved outcomes, although the success of surgical interventions depends on the surgeon’s experience. Typically, during parathyroidectomy, peripheral blood samples collected prior to incision, at incision and 5, 10 and 15-min post incision where a 50% decline in PTH indicates successful adenoma resection. Conventionally, results for samples sent to the clinical laboratory are not usually available before all samples are collected and often take up to 60 min from the initial collection. During this time the patient is on the operating table and surgical team awaiting the outcome of the PTH tests. Therefore, availability of PTH results at the point of surgery with a rapid turn-around time for each sample collection will allow surgeons to make immediate informed decisions. This technology has the potential of significantly reducing surgery and anesthesia exposure times. Additionally, a reliable method of PTH measurement is key for the detection of patients with hyperparathyroidism along with successive follow-up monitoring of medical interventions. READ platform fits perfectly in the clinical workflow to allow surgeons to make decisions regarding the success of the surgery using low sample volume without waiting for time consuming results from the laboratory. Therefore, READ platform is designed to report rapid, accurate and sensitive PTH results addressing the shifting needs and trends of parathyroid surgery in a clinically feasible manner. With an intention to aid surgeons in making rapid informed decisions, an electrochemical READ platform to measure PTH was developed and validated with clinical rigor. This work evinces the importance of determining concordance between laboratory obtained values to READ platform measurements. Moreover, some of the major advantages of READ platform over existing laboratory techniques are as follows (i) READ platform’s small form factor diminishes the need for large operational space and reduces storage requirement. (ii) Portable feature of READ platform enhances efficiency as testing can be conducted flexibly at bedside or near the location of patient care. (iii) READ platform eliminates the need for complicated assay preparation steps prior to testing compared to the Roche analyzer making it a lean process (iv) READ platform provides rapid test results with the propensity to expedite clinical decision-making process (v) As most point-of-care testing with READ platform can be initiated at patient bedside and conducted quickly, the potential for sample deterioration can be reduced drastically.

PTH levels from 40 patient samples were compared between READ platform and commercially available reference standard (Roche analyzer). The READ platform demonstrated good correlation (r = 0.99) with Roche analyzer than the previously reported study (r = 0.93)^25^. Bland Altman analysis showed a good overall agreement between the two methods with a mean bias of 7.6 pg/mL. PTH assays for clinical use have to offer wide dynamic range to encompass the entire physiological range. For instance, PTH levels rise above ~ 500 pg/mL during hyperfunctioning adenoma while post-surgical excision of the hyperfunctioning gland, PTH levels may drop upto ~ 20 pg/mL. The READ platform demonstrates a wide dynamic range from 1 to 1000 pg/mL which accommodates the diverse PTH levels across patients. Additionally, an important consideration when measuring PTH is the presence of cross reacting fragments and other hormones with molecular similarities^26^. Successful PTH assay should possess least cross reactivity to minimize the chances of false positive results. Previous studies have shown variability amongst several commercially available PTH assays that demonstrated varying outcomes^27,28^. This can be attributed to the presence of varying PTH fragments and the presence of structurally similar molecules such as PTHrp in circulation. Predominantly, the liver metabolizes and cleaves most of the (1–84) PTH between amino acid 33 and 36^29^. Once PTH is metabolized, only the quiescent C-terminal segment of PTH (cPTH), consisting of 35 to 84 amino acids, are released back into circulation^30^. Since, the kidney is responsible to clear the inactive cPTH, patients with kidney dysfunction have elevated cPTH fragments^31,32^. This may result in inaccurate results for assays that detect (1–84) PTH. Biological activity is retained by the N-terminal products of PTH metabolism which comprises of first 34 amino acids. To minimize interference from other PTH fragments, we used a highly specific monoclonal antibody directed against the 1–34 bioactive fragment of the PTH molecule. The capture probe functionalized on READ platform is specific to the first 34 amino acid sequence on the amino-terminal end of PTH molecule and does not react with amino-truncated fragments. Furthermore, READ platform was evaluated for interference with PTHrp, Cortisol and ACTH that either have similar structural homology or they are likely to be significantly elevated intraoperatively. The assay showed no cross reactivity (Fig. 7B) with PTHrp nor ACTH, or cortisol. The assay can be linearly operated from 50 to 500 pg/mL. Additionally, high degree of precision was achieved to facilitate reliable treatment following tumor resection. Imprecision studies confirmed the reproducibility of the assay with a variability (%CV) < 10.5%. This level of precision facilitates reliable detection to capture the decline within high PTH levels intraoperatively post tumor resection. The developed READ platform demonstrates rapid results (< 15 min), a feature that is important when being used intraoperatively. READ does not require any sample dilution thereby, reducing sample processing and handling time. READ is a simple-to-use, handheld electrochemical sensing platform that makes it suitable for intra-operative use. It also allows for immediate further exploration of the resection site if the PTH results did not decline appropriately.

In conclusion, the developed READ platform for the measurement of PTH was evaluated for clinical utility. The wide dynamic range detection capability of READ to report significantly elevated PTH levels seen in patients suspected of hyperparathyroidism can be categorized as candidates for parathyroid surgery. READ also exhibited good precision across the measurement range and had good accuracy performance which can help to capture low PTH levels post-surgical excision as an effective POST device. Developing rapid POST devices is not aimed to replace clinical laboratory services but supplement them.

## Materials and methods

### Analytical validation

#### Chemicals and reagents

Dithiobis succinimidyl propionate (DSP) was procured for Pierce Biotechnology. Dimethyl sulfoxide (DMSO) solvent, phosphate Buffer Saline (PBS), SuperBlock along with specific PTH antibody and antigen was obtained from Thermo fisher Scientific. Cortisol for cross reactivity study was procured from Abcam, whereas Parathyroid hormone related protein (PTHrp) and Adrenocorticotropic hormone (ACTH) were obtained from Fitzgerald Industries.

#### Sample collection and preparation

Leftover, de-identified discard samples (heparinized plasma) (n = 40) were obtained from Clements University Hospital (CUH) and Parkland Memorial Hospital (PMH) from patients being investigated for thyroid dysfunction and undergoing parathyroid surgery between September 2018 & July 2019. The study design and experimental protocols were approved by the University of Texas Southwestern Medical Center Institution Review Board (IRB) number 2020-0373 under which, the requirement for patient consent was waived. All the methods were carried out in accordance with clinical guidelines and regulations. The samples were stored at − 20 °C until further use. Samples did not undergo > 2 freeze/thaw cycles prior measurement. PTH levels were measured using both Roche diagnostic Cobas analyzer (reference standard) and READ platform.

#### Rapid electro-analytical device (READ)

The READ point-of-care platform includes a disposable sensor mounted on a handheld electronic reader as explained in detail previously from our group^33^. The sensor surface comprises of a zinc oxide semiconducting layer deposited onto gold electrodes. Fluid confinement on the sensing platform was achieved by fabricating a waterproof silicone barrier using Loctite clear silicone sealant along boundaries of the sensor platform. The assay protocol followed was similar to our previous^12^. Briefly, immunoassay was built on the sensing surface by functionalizing 40 µL of 10 mM Dithiobis (succinimidyl propionate) (DSP) (which is a thiol crosslinker) incubated for 90 min in dark at room temperature. Specific monoclonal Parathyroid Hormone (PTH) antibody and antigen (Thermo fisher) was used to build the calibration curve for the sensor. 40 µL of 10 µg/mL PTH antibody was incubated for 120 min at room temperature. Post antibody immobilization, the sensor was incubated with SuperBlock for 15 min to avoid nonspecific binding interaction. Phosphate buffer saline (PBS) wash was used to remove the unbound molecules and baseline measurement was taken. The sensor was functionalized in the same manner until this assay step followed by the validation experiments as explained in the following sections. READ platform measures the impedance change at the electrode-solution interface when the target PTH binds to immobilized capture antibody by electrochemical impedance spectroscopy (EIS) technique when a small input voltage (10 mV) is applied to the electrode surface.

#### Assay performance characteristics

READ platform performance was assessed with laboratory-based analyzer for functional sensitivity, accuracy, precision, specificity, interference and correlation as follows:

#### Functional sensitivity

Sensitivity was determined following serial dilution of a patient sample with PTH concentration of 493 pg/mL. Doubling dilutions were prepared using pooled plasma as a diluent. Samples were measured in triplicate. Functional sensitivity was determined as the lowest PTH concentration that can be measured with an imprecision of less than 20%.

#### Accuracy

Recovery experiments were conducted to determine the accuracy of the assay. Plasma sample procured from innovative research was spiked with PTH concentrations at 50, 250, 500 and 1000 pg/mL. Prepared samples were measured in triplicates.

#### Precision

Inter-assay and intra-assay imprecision for READ platform for the PTH analyte in plasma was evaluated using low (38.2 pg/mL) and high (942 pg/mL) levels. Each level was analyzed in duplicates with READ platform for a total of 5 days, with a total of 20 samples. Intra-assay, Inter-assay and total imprecision was calculated to identify READ platform precision.

#### Specificity

Effect of interferants in plasma was performed to measure specificity of READ platform. Cross-reactive mixture includes cortisol, Parathyroid hormone related protein (PTHrp), and adrenocorticotropic hormone (ACTH) spiked in plasma with varying concentrations in three different ranges (i) low (200 pg/mL per molecule) represented as D in Fig. 7B, (ii) medium (400 pg/mL per molecule) represented as E in Fig. 7B and (iii) high (1000 pg/mL per molecule) represented as F in Fig. 7B. A schematic representation has been included for better visualization in Fig. 7B.

### Statistical analysis

The data was analyzed and graphed using GraphPad Prism 7(GraphPad Software, Inc.) and Origin. Statistical analysis includes correlation, linear regression and Bland–Altman analysis.

## Supplementary information

Supplementary Information.
