# Supplementary material for: Establish pre-clinical diagnostic efficacy for parathyroid hormone as a point-of-surgery-testing-device (POST)
Source: Sci Rep. 2020 Nov 2;10:18804. doi: 10.1038/s41598-020-75856-2 (PMC7606438; doi:10.1038/s41598-020-75856-2)
Supplement: Supplementary file 1 — Supplementary Information. [file 41598_2020_75856_MOESM1_ESM.docx]

Establish pre-clinical diagnostic efficacy for parathyroid hormone as a point-of-surgery-testing-device (POST)

**Ambalika S Tanak^1^, Sriram Muthukumar^2^, Ibrahim A. Hashim^3^, Shalini Prasad^1^**

1**.** Department of Bioengineering, The University of Texas at Dallas, Richardson,TX, USA 75080

2. EnLiSense LLC, 1813 Audubon Pondway, Allen, TX, USA 75013

3. Department of Pathology, UT Southwestern Medical center, Dallas, TX, USA 75390

**Corresponding Author**: Shalini Prasad

**Address:** 800 W. Campbell Rd. BSB 11,

Richardson, TX, USA 75080

**Email:** [shalini.prasad@utdallas.edu](mailto:shalini.prasad@utdallas.edu)

**Phone**: 972-883-4247

Supplementary


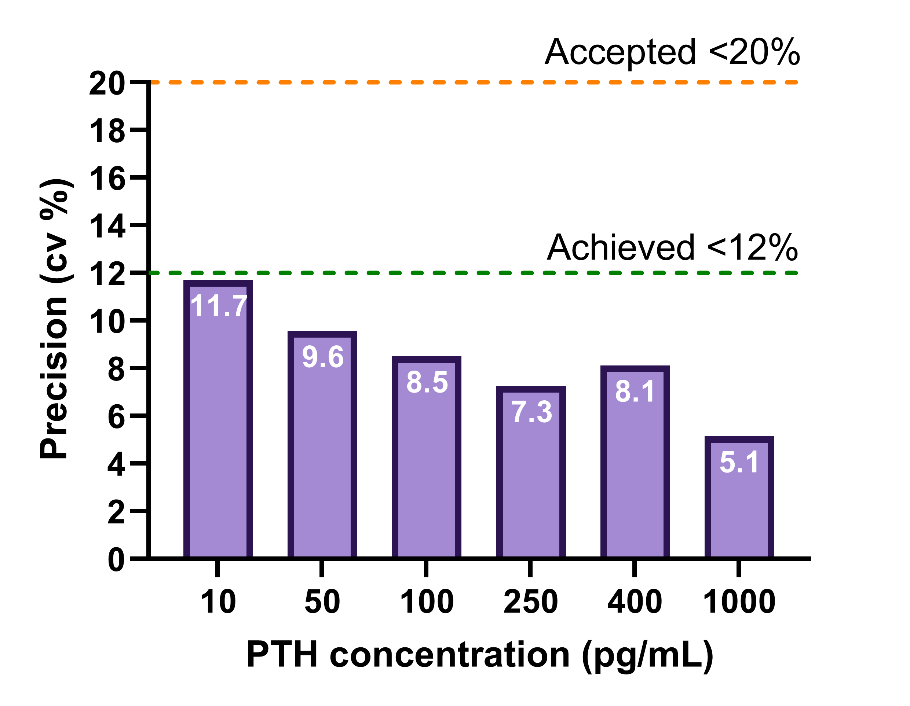
Supplementary figure 1 represents the coefficient of variation (CV) in terms of percentage plotted against each concentration across 6 replicates. CV represents the ratio of standard deviation to the mean within the data set, comparing the degree of variation in different groups. The above figure shows 12% CV for lowest concentration of 10 pg/mL which lies well within the acceptable limit of 20 %, as per CLSI. This demonstrates the ability of READ platform to measure PTH concentration with good precision.

**Table 1 tabulating the spiked and recovered concentrations measured with READ platform.**

| Spiked PTH concentration (pg/mL) | Avg Recovered concentration (pg/mL) | Recovery % |
| --- | --- | --- |
| 10 | 10.2 | 101.7 |
| 50 | 52.6 | 105.2 |
| 100 | 112.4 | 112.4 |
| 250 | 259.0 | 103.6 |
| 400 | 418.4 | 104.6 |
| 1000 | 1004.9 | 100.5 |





Supplementary figure 2 represents pre-calibrated dose response equation used to calculate the PTH concentrations for this study.

| Model | Logistic5 |
| --- | --- |
| Equation | y = Amin + (Amax - Amin) / (1 + (x0/x)^h )^s; |
| Plot | Impedance(ohms) |
| Amin | -37293.67355 ± 8153736.12337 |
| Amax | 6021.3513 ± 338.60489 |
| x0 | 99.64548 ± 2772.74378 |
| h | -0.74773 ± 2.35532 |
| s | 0.02973 ± 6.10641 |
| Reduced Chi-Sqr | 13441.69627 |
| R-Square (COD) | 0.99619 |
